# Safety and Efficacy of Rivaroxaban for Extended-Phase Anticoagulation of Patients with Unprovoked or Recurrent Venous Thromboembolism: Real-Life Data from the MAC Project

**DOI:** 10.3390/life12101657

**Published:** 2022-10-20

**Authors:** Cristiano Bortoluzzi, Enrico Bernardi, Giuseppe Camporese, Franco Noventa, Davide Ceccato, Chiara Tonello, Ngoc Vo Hong, Elena Campello, Chiara Simion, Egidio Imbalzano, Pierpaolo Di Micco, Elena Callegari, Paolo Simioni

**Affiliations:** 1 UO Internal Medicine San Giovanni e Paolo Hospital, 30121 Venice, Italy; 2 Emergency Department ULSS2 “Marca Trevigiana”, 31100 Treviso, Italy; 3 General Medicine, Azienda Ospedaliera, 35100 Padova, Italy; 4 QUOVADIS No-Profit Association, 35139 Padova, Italy; 5 UO Angiology, Azienda Ospedaliera, 35122 Padova, Italy; 6 Thromboembolic Disease Unit Azienda Ospedaliera, 35128 Padova, Italy; 7 Department of Clinical and Experimental Medicine, Polyclinic Hospital University of Messina, 98124 Messina, Italy; 8 Internal Medicine, ASL NA2 Nord-“A. Rizzoli” Polyclinic Hospital, Ischia, 80122 Napoli, Italy; 9 Internal Medicine, Ca’ Foncello Hospital, 31100 Treviso, Italy

**Keywords:** venous thromboembolism, long-term anticoagulation, rivaroxaban

## Abstract

Venous thromboembolism (VTE) is a major cause of death in the world. After the acute-phase treatment, the optimal duration of anticoagulation is still debatable. The latest guidelines suggest maintaining long-term anticoagulation in patients with cancer-associated thrombosis (CAT) or with unprovoked VTE and a low bleeding risk. **Methods:** The MAC Project is an ongoing prospective-cohort, multi-center, observational study in Italy. The project aims to collect real-life clinical information in unselected patients given oral anticoagulants for VTE over a 5-year follow-up period. There were no exclusion criteria, except for life expectancy <6 months and refusal to sign the informed consent form or to attend the planned follow-up visit. All patients were followed-up prospectively with clinical controls scheduled at 3, 6, and 12 months after the index event, and then annually for up to 5 years. The primary efficacy and safety outcomes were symptomatic recurrent VTE and major bleeding. **Results**: We analyzed 450 consecutive patients treated with rivaroxaban and referred them to the MAC Project database for unprovoked or recurrent VTE. Of these, 267 (55%) were unprovoked VTE, and 377 (87%) were symptomatic. We followed up with the patients for a mean of 22 months (Q1 10.7; Q3 37.4 months). Recurrent VTE occurred in 12 patients on rivaroxaban treatment (IR 1.7 per 100 person-years). Males had more recurrence than women. During the follow-up period, we recorded 13 (2.9%) major bleeding, 12 (2.7%) clinically relevant non-major bleeding, 8 minor bleeding, and no fatal bleeding events. Overall, bleeding events occurred in 33 (7.3%) patients, most occurring within the first 2 years of treatment. In addition, we observed a statistically significant higher incidence of bleeding in patients with a baseline HAS-BLED score of 3 to 4 compared with those with a score of 0 to 2, with most events occurring during the first 3 months of treatment (RR 5.9). **Discussion:** Rivaroxaban appears to be safe and effective for the long-term treatment of patients with recurrent or unprovoked VTE. Our results match previously published data, and we are confident that the continuation of the follow-up for up to 5 years will confirm these outcomes.

## 1. Introduction

Venous thromboembolism (VTE) is a major cause of death worldwide [1,2,3]. In patients with unprovoked VTE, the risk of recurrence after treatment suspension is 10% at 1 year, 16% at 2 years, up to 25% at 5 years, and up to 36% at 10 years [4]. Indeed, current guidelines suggest extended-phase anticoagulation in patients with cancer-associated thrombosis (CAT) or unprovoked or recurrent VTE, provided the bleeding risk is acceptable [4,5,6,7,8]. Conversely, extended-phase anticoagulation in patients with a first unprovoked episode of distal deep-vein thrombosis (DVT) is not as mandatory, considering the low (2%) recurrence risk at one year after treatment withdrawal [4,5,6].

The EINSTEIN-Extension study analyzed patients with symptomatic VTE who were randomized to fixed-dose anticoagulation (rivaroxaban 20 mg daily) or a placebo after 6 to 12 months of initial anticoagulant treatment [9]. This study demonstrated the clinical benefit of 2-year extended-phase anticoagulation with rivaroxaban. Indeed, compared with the placebo, treatment with rivaroxaban was associated with a 3-fold reduction in recurrent pulmonary embolism (PE), and with a 7.6-fold decrease in recurrent DVT, without increasing the risk of fatal or major bleeding events [4,10].

A recent cumulative analysis of two large, randomized trials of patients with VTE treated with rivaroxaban confirmed that the recurrence rate is as high as 10% at 1 year in non-anticoagulated patients (placebo group), and as low as 2.0% in patients given rivaroxaban up to 1 year [10]. In turn, one-year treatment with rivaroxaban was safe, the frequency of major bleeding being lower than 1% [10].

Notably, evidence on the efficacy and safety of this approach beyond the second year of treatment is lacking [5,6,7,8,9,10,11,12].

Our study aimed to evaluate the efficacy and safety of rivaroxaban for extended-phase anticoagulation in patients with unprovoked and recurrent VTE included in the MAC (Monitoring Anticoagulant Therapy) Project over a 5-year follow-up period (Clinical Trial Registration number: NCT0432939).

## 2. Study Design

The MAC Project is an ongoing prospective-cohort, multi-center, observational study in Italy. The project aims to collect real-life clinical information in unselected patients given oral anticoagulants for VTE over a 5-year follow-up period. The study protocol, approved by the Institutional Review Board of each participating center, is described in detail elsewhere [13]. Briefly, subjects of both sexes, aged 18 years or older, with objectively diagnosed VT, irrespective of the index event, intended treatment duration, and type of oral anticoagulant treatment, were eligible for inclusion. There were no exclusion criteria, except for a life expectancy <6 months and refusal to sign the informed consent form or to attend the planned follow-up visit. Patients were followed up prospectively at the 3-, 6-, and 12-month visits and then annually for up to 5 years, and the outcomes were verified either at the follow-up visits or otherwise in the patient-reported case. Efficacy was measured by the incidence of symptomatic recurrent VTE, and safety by the incidence of major bleeding, serious adverse events, and mortality.

Patients were not subject to any predetermined intervention but managed according to the clinical practice of each participating clinical center.

Study data were collected and managed using the REDCap electronic data capturing tools, hosted on the QUOVADIS website [14].

In this preliminary report, we describe the outcome of patients with either unprovoked or recurrent VTE at baseline that were given rivaroxaban for at least 3 months and up to 5 years.

## 3. Statistical Analysis

Baseline data were described using standard methods. In addition, Poisson distribution and test-based methods were used to construct the confidence intervals of the incidence rates and the rate ratios.

Cumulative incidences of VTE and bleeding were estimated by the Kaplan–Meier method and compared by various baseline characteristics by the log-rank test.

All the data of the subjects enrolled in the study were used in the outcome analysis. Patients who died or were lost to follow-up were censored at the time of their last examination. In the case of multiple outcomes occurring for one patient, only the first event was counted for the time-dependent analysis. No pre-specified subgroup analysis was planned. All calculations were performed with IBM-SPSS version 26.0 (IBM Corp., Armonk, NY, USA). An alpha error of less than 0.05 was considered significant.

## 4. Results

We analyzed 450 patients with unprovoked or recurrent VTE in the MAC Project database. All patients were given rivaroxaban at the baseline visit. The main patients’ characteristics at enrollment are shown in Table 1. The median follow-up was 22 months (Q1 10.7; Q3 37.4 months). The mean age was 67 years (SD 16.1; Q1 65, Q3 83 years), and 234 (52%) were males. Of those 450 patients, 378 (84%) had a symptomatic index event, 267 (55%) had an unprovoked event, and the remaining 183 (45%) had a VTE recurrence as an index event. The type of index event is detailed in Table 1. Notably, 15 (3%) patients had a previous hemorrhagic event (5 intracranial), 17% had known thrombophilic defects, and 13% used antiplatelet drugs. No patient was lost to follow-up.

### 4.1. Primary Efficacy Endpoint

During the observation period, we recorded 45 (10%) recurrent VTE events; in detail, 12 were proximal DVT, 16 distal DVT, 12 superficial-vein thromboses, and 5 were PE, with 0 fatal events. Of those 45 recurrent events, 12 (incidence rate (IR) 1.7 per 100 person-years, 95% confidence interval (CI) 0.9 to 3.1) occurred while patients were on rivaroxaban and 33 (IR 12.7 per 100 person-years, 95% CI 8.7 to 17.8) in patients who had temporarily or permanently discontinued rivaroxaban (risk ratio (RR) 0.14; 95%CI 0.06 to 0.27; *p* < 0.001). Interestingly, of the 12 recurrences on treatment, 8 (IR 2.2 per 100 person-years, 95% CI 0.9 to 4.3) were recorded in males, and 4 (IR 1.3 per 100 person-years; 95% CI 0.3 to 3.2) in females (RR 1.8, 95% CI 0.5 to 7.9; *p* = 0.35). In turn, of the 33 recurrences out of treatment, 15 (IR 11.3 per 100 person-years; 95% CI 6.3 to 18.6) were recorded in males, and 18 (IR 14.1 per 100 persons-year; 95% CI 8.4 to 22.3) in females (RR 0.8, 95%CI 0.4 to 1.7; *p* = 0.52).

No associations between recurrent VTE and age (>65 years), gender, cancer at baseline, or type of index VTE event were observed (Table 2).

### 4.2. Primary Safety Endpoint

**Figure 1:**
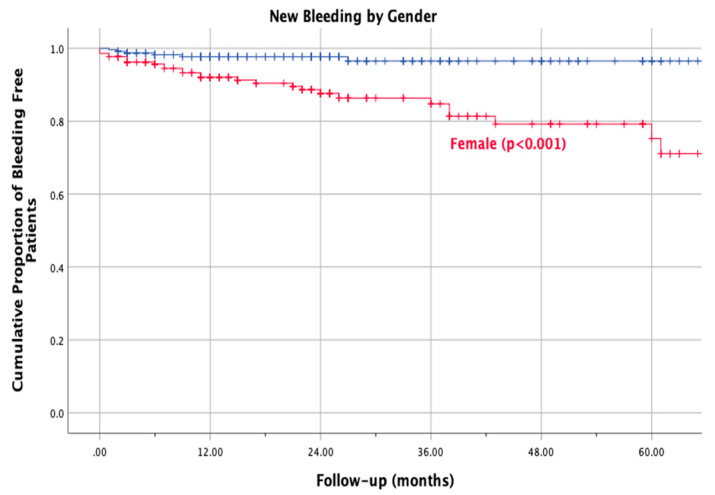
Kaplan-mayer new bleeding by gender.

During the follow-up period, we recorded 13 (2.9%) major bleeding events and 12 (2.7%) clinically relevant non-major bleeding events. We recorded zero fatal bleeding events. Overall, bleeding events occurred in 33 (7.3%) patients; most of them occurred within the first 2 years of treatment. In addition, bleeding events occurred with a statistically significant higher frequency in females than in males (12.5% versus 2.6%; RR 5.0, 95% CI 2.0 to 14.7; log-rank test *p* < 0.001; Figure 1).

**Figure 2:**
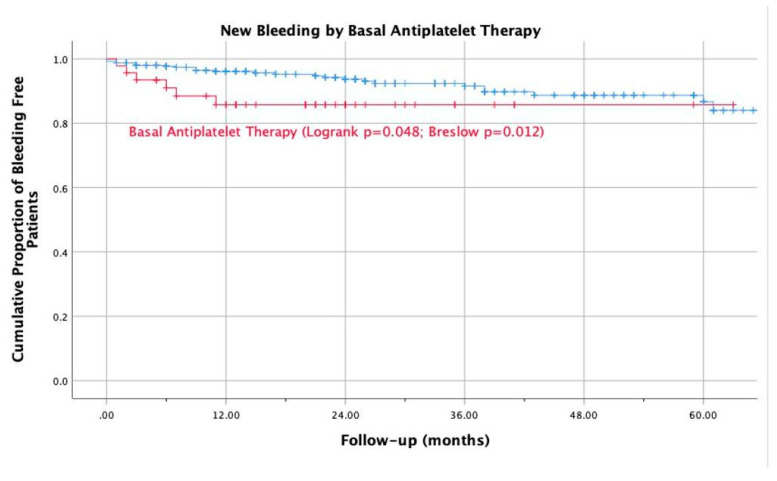
Kaplan Mayer new bleeding in patients taking antiplatelets at baseline vs not taking.

**Figure 3:**
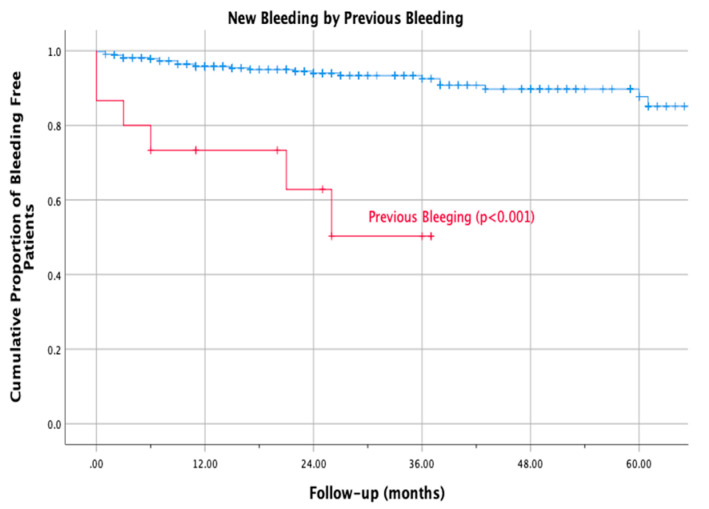
Kaplan Mayer bleeding patients with history of previous bleeding vs non history of previous bleeding.

We also recorded an increase in bleeding events in patients taking antiplatelet drugs as compared to those not on antiplatelets (13% versus 6.7%; RR 2.4, 95%CI 0.8 to 6.0; log-rank test *p* < 0.05; Figure 2); a similar result was found in patients with a history of previous bleeding than in those without (40.0% versus 6.2%; RR 8.8, 95% CI 3.0 to 32.3; log-rank test *p* < 0.001; Figure 3) and in patients with anemia at baseline (Hb < 110 mg/L) versus those without (22.7% versus 6.5%; RR 3.8, 95% CI 1.2 to 10.2; log-rank test *p* = 0.004).

**Figure 4:**
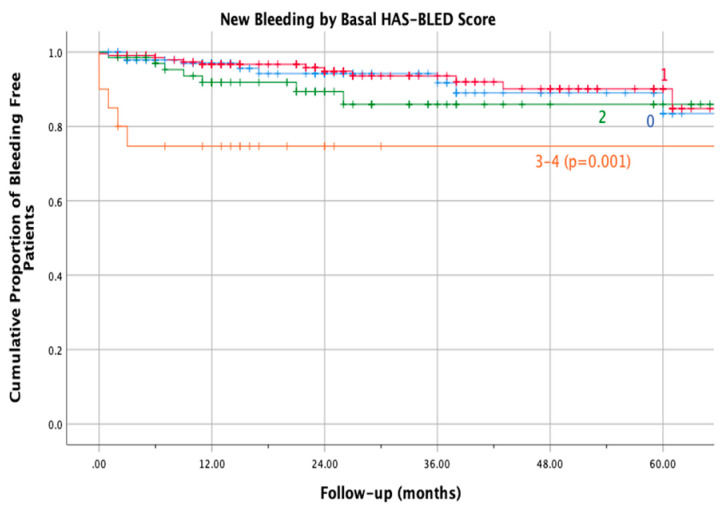
Kaplan Mayer bleeding in different has bleed value.

Furthermore, we observed a statistically significant higher incidence of bleeding in patients with a baseline HAS-BLED score of 3 to 4 as compared to those with a score of 0 to 2 (Table 1), with most events occurring during the first 3 months of treatment (RR 5.9, 95% CI 1.8 to 15.6; log-rank test *p* < 0.001; Figure 4). Specifically, the frequency of bleeding was 6.2% in patients with a HAS-BLED score of 0 (IR 3.2 per 100 person-years; 95% CI 1.5 to 6.0); 5.6% in patients with a score of 1 (IR 2.7 per 100 person-years; 95% CI 1.4 to 4.6); 10.3% in those with a score of 2 (IR 5.0 per 100 person-years; 95% CI 2.0 to 10.3); and 25% in patients with a score of 3 or 4 (IR 19.0 per 100 person-years; 95% CI 6.1 to 44.4).

Two patients with CAT died due to the progression of their malignant disease; no bleeding events occurred in these patients.

## 5. Discussion

Currently, no data from a real-world setting are available on the efficacy and safety of 5-year extended-phase anticoagulation with rivaroxaban in patients with unprovoked or recurrent VTE. Indeed, published data from randomized controlled trials or real-life studies span a maximum follow-up of 4 years [5,8,15].

Of 450 patients with unprovoked or recurrent VTE included in the MAC Project at the moment of the present analysis, more than 60% received more than 2 years of treatment, and around 20% completed the expected 5-year course, the median follow-up being 22 months (Q1 11, Q3 37 months).

On-treatment recurrence occurred with a similar rate to that reported in a recent metanalysis (IR 1.7 vs. 1.5 per 100 person-years, respectively) [16,17], aligning with registered randomized trials (Einstein DVT, PE, and Ext). Extended-phase treatment with rivaroxaban was associated with a statistically significant reduction in recurrent events. Notably, the frequency of recurrent VTE in patients who had temporarily, or permanently discontinued rivaroxaban was higher than in the literature (13% vs. 8%) [17]; however, the high proportion of patients with distal-vein DVT in our cohort (Table 1) could have an impact on this outcome.

Furthermore, on-treatment recurrences were recorded more frequently in males, a finding already well documented but still hardly explained [17,18].

Overall, the frequency of bleeding events was slightly lower than that reported in a recent meta-analysis [19]; however, it was higher than that observed in registered studies, most likely because the latter excluded patients at higher bleeding risk or with anemia at baseline [9,11].

We observed an association between bleeding events and a history of previous bleeding (OR 8.1 *p* < 0.001) or anemia (OR 4.5 *p* < 0.001) at enrollment. Such findings confirm what was recently reported by a meta-analysis of all trials investigating direct anticoagulant use for VTE treatment [19], although the follow-up in the latter was limited to 12 months. They are also in line with observations from the RIETE Registry, although in patients treated only with VKA [15,20,21,22,23].

We also found a correlation between bleeding events and the HAS-BLED score at enrollment, as already reported in the literature [20]. This was predictable, as the HAS-BLED score incorporates both a history of major bleeding (+1 point) and the use of medications predisposing to bleeding (+1 point), two conditions that were significantly associated with bleeding in our cohort (Table 2).

Based on these observations, we believe that patients with a HAS-BLED higher than 2 should be treated but carefully monitored, especially during the first months of anticoagulation, due to the high risk of bleeding.

Our study has some limitations. First, this is not a randomized study and therefore lacks a control group; however, there were no patient selections at the enrollment, and they were entered in the database consecutively. These are the characteristics of a real-life study and represent a picture of the real-world use of rivaroxaban. Nonetheless, we used a robust methodology, and we do not have any patients lost to follow-up. Secondly, the number of patients who completed the expected 5-year follow-up period is still small, but this is due to our database’s “youth” problem—enrollment started five years ago and is still ongoing. Nonetheless, our preliminary data seem to align with what is already described in the literature.

In conclusion, rivaroxaban appears to be safe and effective for the long-term treatment of patients with recurrent or unprovoked VTE. Of note, though preliminary, our results match with previously published data and potentially expand our confidence in a long-term treatment span of 5 years.

## Figures and Tables

**Table 1:** The patients’ main characteristics at baseline.

Index Event	n	%
Deep-vein thrombosis		
Proximal	163 *	36.3
Distal	195 †	43.3
Pulmonary embolism	21	4.7
Deep-vein thrombosis and pulmonary embolism	71	15.7
Medical conditions	n.	%
Hypertension (>140 mmHg or in therapy)	240	53.6
Diabetes	39	8.7
COPD	14	3.1
Recent infection (<3 months)	18	4.0
Previous stroke	18	4.0
Congestive heart failure	9	2.0
Peripheral arterial disease	33	7.4
Varicose veins	160	35.6
Previous venous thromboembolism	233	52.0
Renal dysfunction (eGFR <30 mL/min)	15	3.3
Dislipidemia	80	17.8
Active cancer	28	6.2
Autoimmune disease	36	8.0
Esophagitis, gastritis, inflammatory bowel disease	50	11.1
Hormone therapy	4	0.9
Active smoking	63	14.0
Known thrombophilia	76	16.9
Protein c deficiency	9	2.0
Protein s deficiency	6	1.3
Factor V leiden. Apc resistenza	43	9.5
Prothrombin variant G20210A	13	2.9
Antiphospholipid syndrome	4	0.9
Previous hemorrhagic events	15	3.3
Intracranial	5	1.1
Gastro-intestinal	4	0.7
Medication Use	n.	%
Low-dose aspirin	46	11.2
P2Y12 inhibitor	6	1.3
Double antiaggregation	3	0.7
Antihypertensive treatment	61	13.6
Oral antidiabetics	34	7.6
Hypolipidemic drugs	86	19.1
NSAIDS	6	1.3
Laboratory values	mean	SD
Hemoglobin g/L	138.1	44.7
Platelets 100 × 10^6^/L	229.8	67.5
Serum creatinine (mL/min)	38.9	44.7p
Creatinine clearance (Cockcroft–Gault formula) mL/min	75.9	32.8
HAS-BLED score	n	%
0	146	32.4
1	214	47.6
2	68	15.1
3–4	22	4.9

* Including 12 (2.7%) upper limb DVT; † Isolated muscular (gastrocnemius or sural) distal DVT or axial distal DVT (peroneal, anterior tibial, posterior tibial), or both.

**Table 2:** Interactions between the patients’ characteristics at baseline and VTE recurrence or bleeding.

			Recurrent VTE			Bleeding		
Basal Risk Factors		No.	n. (%)	IR (95% C.I.)	Logrank *p*	n. (%)	IR (95% C.I.)	Logrank *p*
Age	<65a	193	20 (10.4)	5.5 (3.3–8.4)	0.673	13 (6.7)	3.5 (1.8–5.9)	0.762
	≥65a	257	25 (9.7)	4.7 (3.1–7.0)		20 (7.8)	3.8 (2.3–5.8)	
Gender	Male	234	23 (9.8)	4.9 (3.1–7.3)	0.786	6 (2.6)	1.2 (0.5–2.7)	<0.001
	Female	216	22 (10.2)	5.3 (3.3–7.9)		27 (12.5)	6.5 (4.3–9.4)	
Cancer	With	28	2 (7.1)	3.2 (0.4–11.4)	0.471	0	-	0.111
	Without	422	43 (10.2)	5.2 (3.8–7.0)		33 (7.8)	3.9 (2.7–5.5)	
VTE type	Proximal + PE	232	24 (10.3)	4.7 (3.0–7.0)	0.698	24 (10.3)	4.8 (3.1–7.1)	0.085
	Distal	89	8 (9.0)	6.4 (2.8–12.6)		2 (2.2)	1.6 (0.2–5.6)	
Antiplatelet therapy	With	46	7 (15.2)	9.6 (3.8–19.7)	0.107	6 (13)	8.1 (3.0–17.7)	0.048
	Without	404	38 (9.4)	4.6 (3.3–6.4)		27 (6.7)	3.3 (2.1–4.7)	
Previous Bleeding	With	15	1 (6.7)	2.9 (0.1–16.3)	0.505	6 (40)	27.1 (9.9–59.1)	<0.001
	Without	435	44 (10.1)	5.1 (3.7–6.9)		27 (6.2)	3.1 (2.0–4.5)	
Anemia (HB < 110 gr/L)	With	22	3 (13.6)	7.1 (1.5–20.8)	0.553	5 (22.7)	11.9 (3.8–27.7)	0.004
	Without	400	38 (9.5)	4.5 (3.2–6.2)		26 (6.5)	3.1 (2.0–4.5)	

IR: incidence rate (events per 100 persons-year).

## Data Availability

Data were contained within the article.
